# Treating a Case of Class II Caries With Silver Diamine Fluoride: A Newer Approach

**DOI:** 10.7759/cureus.70427

**Published:** 2024-09-29

**Authors:** Mohammad Shafae Azmi, Neha Pankey, Manoj Chandak, Ramakrishna Yeluri, Priyanka D Sontakke, Rahul Ravi

**Affiliations:** 1 Department of Oral Pathology and Microbiology, Sharad Pawar Dental College and Hospital, Datta Meghe Institute of Higher Education and Research, Wardha, IND; 2 Department of Pediatric and Preventive Dentistry, Sharad Pawar Dental College and Hospital, Datta Meghe Institute of Higher Education and Research, Wardha, IND; 3 Department of Conservative Dentistry and Endodontics, Sharad Pawar Dental College and Hospital, Datta Meghe Institute of Higher Education and Research, Wardha, IND; 4 Department of Public Health Dentistry, Sharad Pawar Dental College and Hospital, Datta Meghe Institute of Higher Education and Research, Wardha, IND; 5 Department of Orthodontics, Sharad Pawar Dental College and Hospital, Datta Meghe Institute of Higher Education and Research, Wardha, IND

**Keywords:** dental caries, early childhood caries, enamel remineralization, silver diamine fluoride, stainless steel crown

## Abstract

This case report details the comprehensive management of a six-year-old child diagnosed with early childhood caries (ECC), a prevalent condition affecting young children. The patient presented with acute pain in the lower left quadrant of the jaw, specifically involving the deciduous mandibular first molar (tooth D), which exhibited Class II caries. A prompt intervention was necessary to prevent further decaying of the tooth and manage the child's discomfort. The treatment strategy focused on a minimally invasive approach, beginning with the application of silver diamine fluoride (SDF) to arrest the progression of dental caries. SDF is known for its efficacy in halting carious lesions and providing immediate pain relief, making it a suitable option for young patients. Following the stabilization of the carious lesion, a stainless steel crown (SSC) was placed over the affected tooth. This restoration not only preserved the structural integrity of the tooth but also restored the masticatory function and maintained an aesthetic appearance, which is crucial for the child's overall oral health and development. This case highlights the effectiveness of combining SDF treatment with SSC placement in managing ECC, thus providing a durable solution that offers both pain relief and long-term protection against further carious activity. The chosen method underscores the importance of early intervention and the use of child-friendly restorative techniques in pediatric dentistry.

## Introduction

G.V. Black observed something significant in 1900 when he said, "The most common ailment that humans are susceptible to is caries of the teeth. No other illness affects as many members of the human family as this one" [1]. It seems that this statement is still relevant more than a century later, as dental caries is still remarkably common. With an estimated 2.5 billion individuals worldwide suffering from this enduring ailment, untreated dental caries in permanent teeth remains the most common condition impacting humanity today. The problem of controlling and preventing dental caries remains a significant public health concern despite improvements in dental care and public health programs, highlighting the necessity for ongoing efforts and creative solutions [2].

Dental caries ranks as the most prevalent chronic disease affecting children on a global scale, with significant implications for overall health and well-being [3-5]. Early childhood caries (ECC), defined by the presence of one or more decayed, missing, or filled tooth surfaces (DMFS) in any primary tooth of a preschool-aged child, is a specific manifestation of this widespread condition. The Association of State and Territorial Dental Directors recognized ECC as a critical public health concern, highlighting the urgent need for effective prevention and management strategies to address this pervasive issue [6]. In this case report, the subject was a caries-active patient and hence had high susceptibility to secondary caries. Also class II caries shows increased probability of proximal fracture. Silver diamine fluoride (SDF) is a clear, odorless solution containing silver, fluoride, and ammonium ions. When applied to carious tooth tissue, its high fluoride content of 44,800 ppm efficiently promotes tooth desensitization and carious lesion arrest through chemical reactions. It works in two ways: the fluoride stops further demineralization of the tooth structure, while the silver component functions as an antibacterial agent by killing the bacteria and preventing the production of new biofilm [7,8,9]. One noteworthy adverse effect though is that sound enamel is unaffected, while carious lesions carry a permanent black stain [10]. This case demonstrates the success of using SDF treatment alongside SSC placement to manage ECC, offering lasting pain relief and protection against future caries. It emphasizes the value of early intervention and child-friendly restorative methods in pediatric dentistry.

## Case presentation

**Figure 1:**
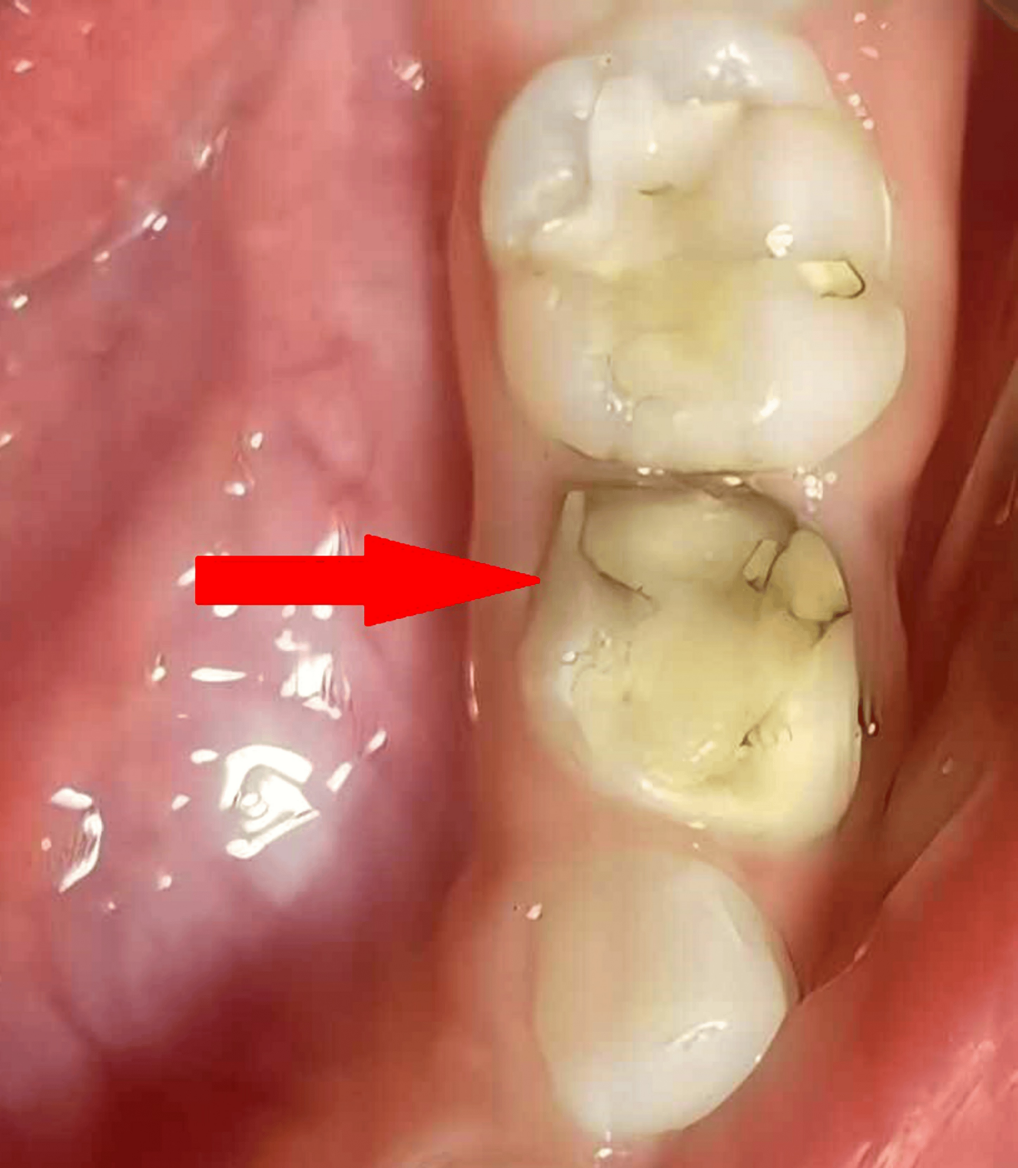
Class II caries (indicated by a red arrow) in the lower left first primary molar

A six-year-old male presented with a chief complaint of pain in the lower left back tooth region of his jaw. The pain had been persistent for several days, affecting his ability to eat and sleep comfortably. His medical history was unremarkable with no known allergies or systemic conditions. The past dental history of the patient reveals multiple restorations of teeth in both maxillary and mandibular arches. Figure 1 shows class II caries in the lower left first primary molar.

The clinical examination revealed extensive caries on the lower left primary mandibular first molar (D), specifically a class II lesion involving the proximal surface extending into the dentin. The tooth was non-tender to percussion, suggesting no pulpal involvement. Also, there were no signs of swelling or abscess formation. Considering all the signs and symptoms, a diagnosis was made, which was early childhood caries. Given the diagnosis of ECC with class II caries in the lower left primary mandibular first molar tooth D, a treatment plan was developed to address both immediate pain relief and long-term tooth preservation. As previous restoration of the tooth done using glass ionomer cement (GIC) had a poor prognosis, to arrest the caries progression, 38% silver diamine fluoride (SDF) was applied to the affected proximal and occlusal surfaces of tooth. The parent of the child was informed about the potential for the carious lesion to turn black, which is a normal and expected outcome of the SDF application. After ensuring the SDF had adequately dried, the tooth was prepared for a stainless steel crown (SSC). SSC was modified to adequately adapt to the tooth using crown crimping plier and Howe plier. The SSC was then fitted and cemented over tooth D, providing a durable and protective restoration. The choice of silver crown was based on its effectiveness in restoring function and preventing further decay in primary teeth.

**Figure 2:**
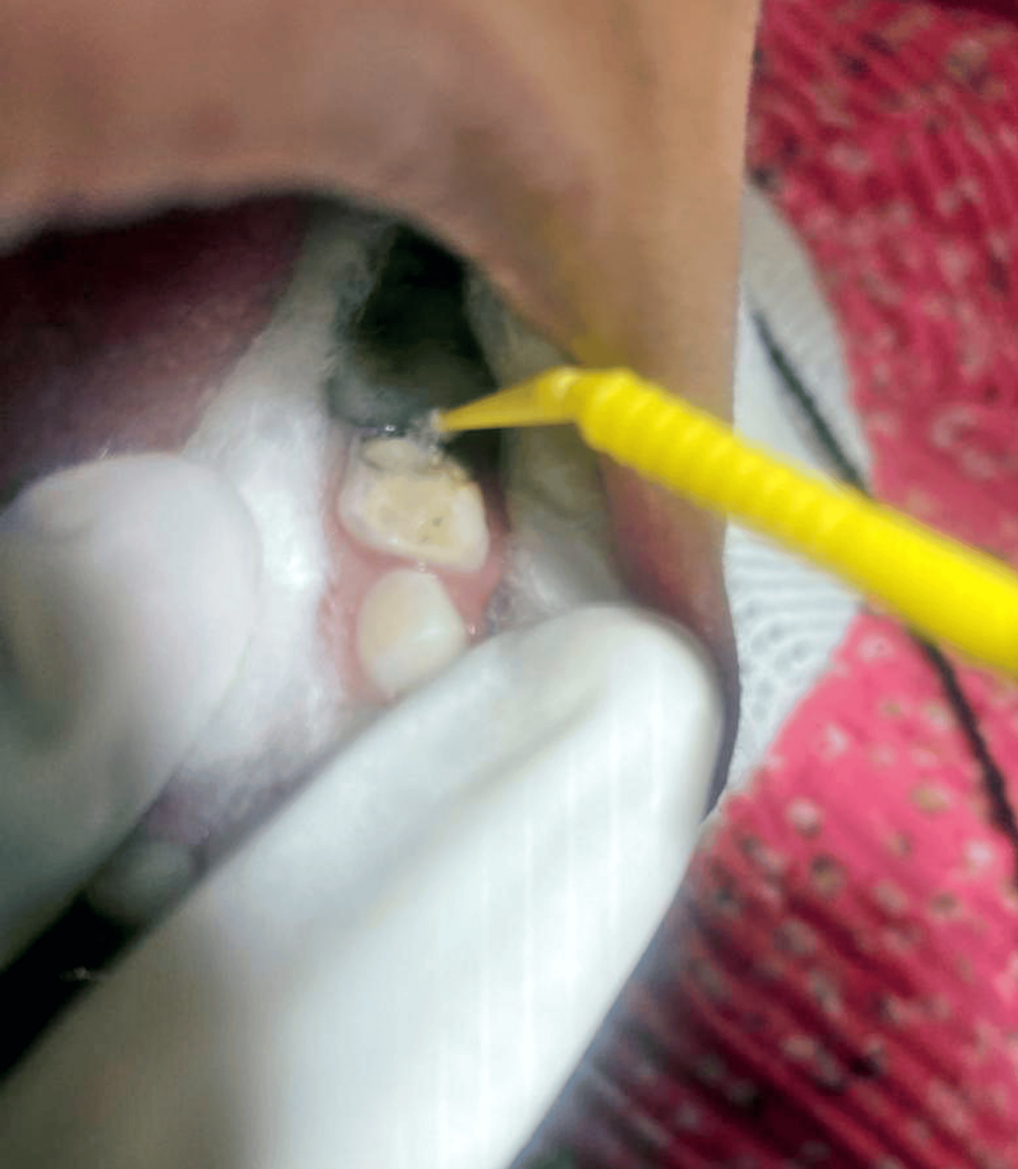
Application of silver diamine fluoride with microbrush applicator tip

**Figure 3:**
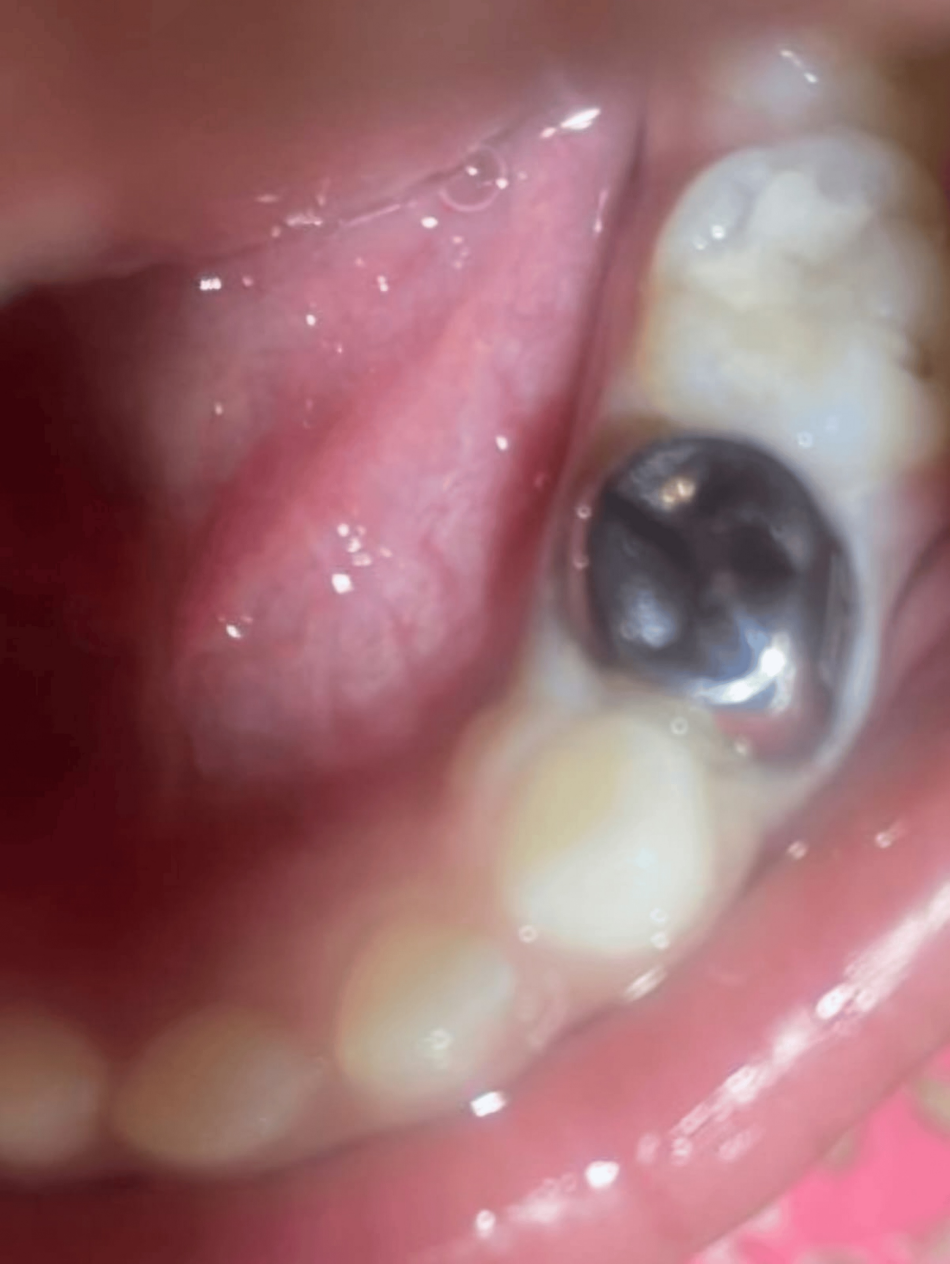
Stainless steel crown application

Instructions were given to the parent on after-therapy precautions. Guidance was provided on how to take proper care of one’s mouth such as avoiding sugary drinks and foods, among others, and twice daily brushing using toothpaste with fluoride. The importance of having dental check up every six months to monitor the condition of SSC as well as oral health at large was also stressed. Figure 2 shows the application of SDF with the help of applicator tip and Figure 3 displays the stainless steel crown application.

The patient was assessed in a follow-up appointment one month after the procedure. During the follow-up, the patient did not report any additional pain, and the SSC was still in place and functioning normally. The success of the treatment therefore could be confirmed by seeing symptoms being alleviated as well as long-term effects from the restorative work done.

## Discussion

This example illustrates how ECC may be effectively managed, pain can be relieved, and young patients' dental health can be maintained by combining restorative and preventative care. While the SSC provided a long-lasting repair that safeguarded the tooth from additional decay and kept its function, the use of SDF allowed for non-invasive caries arrest. Because of their adhesion to the tooth structure, minimal polymerization contraction, absence of postoperative sensitivity, biological compatibility, and fluoride release's anti-cariogenic properties, GICs are suitable materials for primary dentition [11]. However, with a failure rate of 6.6%-30% after 36 months, their poor resilience to wear and fracture renders them unsuitable as materials for proximal restorations [12].

Chu et al. (2012) [13] and Richards (2017) [14], in their study, concluded that several pieces of data indicate that SDF slows the advancement of caries and speeds up the remineralization process of enamel. It has also been shown that the use of SDF lessens the need for extensive restorative care in kids, which in some cases could otherwise necessitate general anesthesia [15]. SDF was utilized to stop the progression of caries and limit the chance of crown breakage by utilizing a stainless steel crown. As clearly stated above by Chadwick and Evans (2016), GIC has poor resistance to fracture and it was evident that we use SDF with stainless steel crown [12].

Traditional restorative methods for class II caries, such as amalgam or composite resin fillings, have long been favored due to their ability to restore tooth function and aesthetics. However, these procedures often require significant removal of healthy tooth structure to gain access to the carious lesion. In contrast, SDF provides a non-invasive alternative that arrests caries without the need for extensive tooth preparation [16]. Several studies have shown that SDF is highly effective in halting the progression of carious lesions, with one study by Gao et al. (2016) [17] reporting a 90% success rate in arresting caries in primary teeth when SDF was applied biannually. Yee et al. (2009) [7] demonstrated that SDF was as effective as traditional restorations in preventing the progression of carious lesions in primary molars over a two-year period.

One of the primary concerns associated with SDF application is the black staining that occurs on treated carious lesions. This cosmetic drawback has been a significant barrier to its widespread adoption, especially in anterior teeth or in patients who prioritize aesthetics. However, studies have shown that patient and caregiver acceptance of SDF is high when the benefits are clearly explained. According to a study by Crystal et al. (2017), most parents accepted SDF treatment for their children despite the staining, especially when the alternative was a more invasive procedure [18]. Verbal and written consent from the parent was taken prior to the commencement of the treatment after discussing the pros and cons of SDF versus traditional restorations.

## Conclusions

In this study, the treatment for a six-year-old's early childhood caries (ECC), which included using a stainless steel crown and silver diamine fluoride (SDF), was very successful. This technique arrested the caries from spreading, alleviated the child's suffering, and restored the tooth's function in a long-term and less invasive method. Because the treatment was minimally invasive, it also made managing the child's behavior during the procedure easier. Regular check-ups and preventive care are crucial for children to keep their teeth healthy over time.
